# Provable randomized rounding for minimum-similarity diversification

**DOI:** 10.1007/s10618-021-00811-2

**Published:** 2022-01-04

**Authors:** Bruno Ordozgoiti, Ananth Mahadevan, Antonis Matakos, Aristides Gionis

**Affiliations:** 1 Department of Computer Science, Aalto University, Espoo, Finland; 2 Department of Computer Science, University of Helsinki, Helsinki, Finland; 3 Division of Theoretical Computer Science, KTH Royal Institute of Technology, Stockholm, Sweden

**Keywords:** Diversification, Recommender systems, Randomized rounding, Quadratic programming

## Abstract

When searching for information in a data collection, we are often interested not only in finding relevant items, but also in assembling a diverse set, so as to explore different concepts that are present in the data. This problem has been researched extensively. However, finding a set of items with minimal pairwise similarities can be computationally challenging, and most existing works striving for quality guarantees assume that item relatedness is measured by a distance function. Given the widespread use of similarity functions in many domains, we believe this to be an important gap in the literature. In this paper we study the problem of finding a diverse set of items, when item relatedness is measured by a similarity function. We formulate the diversification task using a flexible, broadly applicable minimization objective, consisting of the sum of pairwise similarities of the selected items and a relevance penalty term. To find good solutions we adopt a randomized rounding strategy, which is challenging to analyze because of the cardinality constraint present in our formulation. Even though this obstacle can be overcome using dependent rounding, we show that it is possible to obtain provably good solutions using an independent approach, which is faster, simpler to implement and completely parallelizable. Our analysis relies on a novel bound for the ratio of Poisson-Binomial densities, which is of independent interest and has potential implications for other combinatorial-optimization problems. We leverage this result to design an efficient randomized algorithm that provides a lower-order additive approximation guarantee. We validate our method using several benchmark datasets, and show that it consistently outperforms the greedy approaches that are commonly used in the literature.

## Introduction

In this paper we consider the problem of *diversified item selection*, which is ubiquitous in search and recommendation scenarios. In the simplest embodiment of item selection problems, one is given a query and must then search the available database for the most relevant results. In web search, for example, users input a set of keywords, and the search engine must produce the documents that most strongly relate to those keywords. However, in the absence of adequate measures, the top results might be too similar to each other, and thus only one of them might be of interest to the user. Therefore, it is often desirable to select a number of items that are not only relevant to the given query, but also not too similar to each other, so as to avoid redundant results.

We formalize the problem of selecting *k* diverse items from a collection of *n* items as that of *minimizing* an expression of the form $\mathbf {x}^T\mathbf {W}\mathbf {x}+\mathbf {c}^T\mathbf {x}$ such that $\mathbf {x}\in \{0,1\}^n$ and $\sum _i x_i=k$. The $n\times n$ non-negative matrix $\mathbf {W}$, which is assumed to have a zero diagonal, models pairwise item similarity and $\mathbf {c}$ represents a relevance-loss vector. This formulation transcends diversification and encompasses a wide array of problems, such as *k*-independent set, quadratic knapsack or $k$ -Sparsest-Subgraph, and is thus of special interest in combinatorial optimization.

Solving programs of this type is $\mathbf {NP}$-hard in general, even when the variables are relaxed to the convex set $[0,1]^n$ (Vavasis 1992). Polynomial-time approximation schemes are possible when the matrix in the quadratic term has at most one positive or one negative eigenvalue (Hildebrand et al. 2016). In the optimization literature, the focus has been chiefly on reformulations to leverage the existence of highly-optimized solvers, such as convexification, linearization and semidefinite relaxations (Carter 1984; Zhang 2000; Billionnet et al. 2008; Lima and Grossmann 2017), or in the development of branch-and-bound methods to obtain exact solutions (Kalantari and Bagchi 1990). The underlying goal is to obtain methods with good empirical performance. A nice overview can be found in the recent work of Furini et al. (Furini et al. 2019).

Quadratic programs, and perhaps more commonly the special case of linear programs, are also frequently the subject of attention in combinatorial optimization, as many discrete problems can be written in this form. By *relaxing* the feasible set, e.g., to allow solutions $x \in [0,1]^n$, and then *rounding* the result back to $\{0,1\}^n$, it is sometimes possible to obtain a *feasible solution* to the original problem with *provable quality guarantees* (Jain and Vazirani 1999; Charikar and Li 2012).

*Randomized rounding* (Raghavan and Tompson 1987) is a common technique to obtain a discrete feasible solution by solving first a continuous relaxation. The method can be roughly described as follows: each discrete variable is randomly set to 1 with a probability derived from the corresponding entry of the continuous solution to the relaxed progream. When the rounding can be done *independently* for each variable, this process is often easy to analyze and produces solutions with approximation guarantees in expectation (Williamson and Shmoys 2011).

*Rounding under cardinality constraints* Applying this technique to the problem we consider in this paper is challenging due to the presence of a cardinality constraint, as the independent nature of the rounding may result in solutions of any size. Some attention has been devoted to obtaining samples respecting a constraint of this nature. Srinivasan proposed a sampling algorithm to ensure the desired cardinality (Srinivasan 2001). Related alternatives, including derandomization methods, were later proposed (Gandhi et al. 2002; Doerr 2005). But these approaches come at a cost: the dependent nature of the process makes the algorithms slightly more involved to implement, requiring substantially more coin flips, and hurts parallelizability. In certain scenarios, one might wish to preserve the straightforward simplicity of independent rounding, and the increased running time can be inconvenient in critical applications. Thus, in this paper we aim to answer the following question: *can we use independent rounding and provide provable approximation guarantees in cardinality-constrained settings?*

We answer this question positively. We rely on the fact that a natural relaxation of our program induces a sampling distribution, which concentrates sharply around the mean, and thus we can obtain feasible solutions with high probability after a small number of attempts. The procedure is not straightforward to analyze, as conditioning on feasibility introduces unwieldy factors in the expected value of the solution. We bound these factors by leveraging their symmetry, which allows us to reason about the structure of the extrema. The derived bound applies to ratios of symmetric polynomials, which is of independent interest. In the end we are able to show that, with high probability, we can efficiently obtain a discrete solution of the desired cardinality whose value in the objective is within a small constant of the continuous one. To the best of our knowledge, this is the first result showing an approximation guarantee of independent randomized rounding for the proposed quadratic programs under cardinality constraints. It should be noted that our analysis leaves the simplest embodiment of randomized rounding intact, and is therefore straightforward to implement and perfectly parallelizable. To evaluate the practical advantages of the proposed method, we compare it empirically to an existing method for rounding with cardinality constraints. The results show that our approach achieves significant speed-ups in some regimes, especially when parallelization is possible. Nevertheless, the existing dependent method can be faster in some cases. In the experiments we delve into the details of these differences and discuss pros and cons of each technique.

*Applications* To illustrate the applicability of our result, we address the problem of search-result diversification, when the goal is to minimize pairwise similarities between the retrieved items. Natural formulations of this objective are hard to approximate, and thus the related literature has focused mostly on maximization variants. However, we argue that minimization approaches for diversification are of interest to the information-retrieval community, as similarity functions, instead of distances, are commonly used to compare documents. Furthermore, we now illustrate with an example that methods giving approximation guarantees in maximization formulations can be arbitrarily bad for minimization.

### Example 1

Given a collection of documents, we want to find a set of *k* documents *S* for which the sum of pairwise cosine similarities $\sum _{\mathbf {x},\mathbf {y}\in S, \mathbf {x}\not =\mathbf {y}} \cos (\mathbf {x},\mathbf {y})$ is minimized. Consider a problem instance in which the optimal solution $S^*$ contains *k* documents satisfying $\cos (\mathbf {x},\mathbf {y})=\epsilon$ for all $\mathbf {x},\mathbf {y}\in S^*$. We assume that vectors are normalized, so Euclidean distances $d(\mathbf {x}, \mathbf {y})$ are related with cosine similarities by $d(\mathbf {x}, \mathbf {y})=\sqrt{2-2\cos (\mathbf {x},\mathbf {y})}$. For the documents in $S^*$ all pairwise distances are $\delta =\sqrt{2-2\epsilon }$. Assume that we maximize distance diversity using an algorithm with approximation guarantee $\alpha =\frac{1}{2}$, Thus, the approximation algorithm may return a solution $S'$ in which the average pairwise distances is $\delta '=\frac{1}{2}\delta$. But for a pair at distance $\delta '$, the cosine similarity will be $\sigma '$, so that $\delta '=\sqrt{2-2\sigma '}=\frac{1}{2}\sqrt{2-2\epsilon } \Leftrightarrow \sigma ' = \frac{3+\epsilon }{4}$. Therefore, the cost of the optimal solution $S^*$ is $\frac{k(k-1)}{2}\epsilon$, while this scheme may yield a solution $S'$ with cost approximately $\frac{k(k-1)}{2}\!\cdot \!\frac{3+\epsilon }{4}$, which is arbitrarily bad compared to $S^*$.

Despite the inapproximability of our formulation, we leverage our randomized-rounding analysis to give an efficient algorithm that returns, with high probability, a solution within a lower-order additive term and a small multiplicative constant of the optimum. Our experiments show how this approach consistently outperforms greedy method, which is the conventional approach that has been proposed to tackle similar objectives.

Our approach is a significant step towards bridging a gap between theory and practice for the problem of diverse-item selection. This gap stems from the fact that, on the one hand, similarity functions are widely used in many real-world applications, while on the other hand, the theory of diverse-item selection relies almost exclusively on distance functions.

Contributions In summary, in this paper we make the following contributions:

- We present a novel analysis of independent randomized rounding with cardinality constraints, applied to the minimization of 0-1 quadratic programs. In particular, we show that the rounded solution is within a small constant factor of the given fractional solution.
- We use this result to obtain an efficient algorithm with an additive approximation guarantee for the problem of search-result diversification.
- Our experimental setup demonstrates how this approach outperforms greedy methods, which are the established method to tackle similar objectives.
- We carry out an extensive comparison of the proposed independent rounding method and an existing dependent method.

The rest of this paper is organized as follows. In Sect. 2 we discuss the related work and put our work in context. In Sect. 3 we formally define our problem formulation and establish its hardness. In Sect. 4 we describe independent randomized rounding and analyze it in our context. In Sect. 5 we describe some cases in which good continuous solutions can be found efficiently. In Sect. 6 we show how our result can be used to obtain provably good solutions to a widely applicable formulation of search-result diversification. In Sect. 6 we discuss practical implementation issues and present a scalable sampling-based adaptation of our method. In Sect. 7 we present our experimental evaluation, and in Sect. 8 we offer concluding remarks and point to directions for future work.

## Related work

*Randomized rounding* A long line of research has focused on randomized rounding as a technique to obtain a provably good solution to a 0-1 integer linear program. The technique was introduced by Raghavan and Tompson (Raghavan and Tompson 1987) to derive approximation algorithms for various NP-complete problems. This approach is not suitable for cardinality-contrained problems, as in our formulation, because the entries of the solution vector are rounded independently and thus cardinality can be violated. To address this issue, some works have proposed dependent rounding methods, to respect cardinality as well as certain negative correlation properties useful for proving concentration bounds (Srinivasan 2001; Doerr 2005). Ensuing work has dealt with applications and related problems (Gandhi et al. 2002; Chekuri et al. 2010). We recommend the comprehensive survey of Doerr and Wahlström for an overview of the field (Doerr and Wahlström 2016).

The first method we are aware of for randomized rounding under cardinality constraints (Srinivasan 2001) works by modifying probabilities in pairs, which results in a binary tree whose levels must be processed sequentially. This limits the parallelizability of the approach, requiring ${\varOmega } (\log n)$ sequential steps. A closely related line of work was pursued by Doerr and Wahlström (Doerr 2005; Doerr and Wahlström 2015). They preserve the required cardinality by rounding variable pairs at the bit level, so as to preserve their sum, until all are integral. This entails ${\varOmega } (n\ell )$ coin flips, where $\ell$ is the bit precision required to represent the available variables.

Our approach, in contrast, preserves the simplicity and full parallelizability of straightforward independent rounding. As a trade-off, we require ${\varOmega } (\sqrt{k})$ attempts to ensure a satisfactory solution with constant probability. Nevertheless, these attempts can also be done in parallel.

*Search result diversification* With the Maximal Marginal Relevance (MMR) algorithm, Carbonell and Goldstein were the first to propose a method for diversified search results (Carbonell and Goldstein 1998). Their seminal paper spurred a range of proposals taking different approaches to diversification (Ziegler et al. 2005; Radlinski and Dumais 2006; Clarke et al. 2008; Dou et al. 2011; Santos et al. 2010; Capannini et al. 2011). The works closer to ours in spirit are that of Zhang and Hurley, since they propose maximizing a quadratic objective and rounding to an integral solution (Zhang and Hurley 2008), and Rafiei et al. since they model the relationship between queries and results as a random variable, and then optimize the argument of a quadratic form on the covariance matrix (Rafiei et al. 2010).

More relevant is the line of research that focuses on approximation guarantess. Diversification is closely related to dispersion (Chandra and Halldórsson 2001). Some authors rely on well-known results on the maximization of monotone submodular function subject to cardinality (Nemhauser et al. 1978) and matroid (Calinescu et al. 2007) constraints (Bansal et al. 2010; Tsaparas et al. 2011; Tong et al. 2011; He et al. 2012). Bansal et al. consider *discounted cumulative gain* and per-user subtopic coverage constraints, and give a PTAS for the case where each user requires one topic (Bansal et al. 2010). Borodin et al. give a 2-approximation to a maximization objective similar to ours (Borodin et al. 2017), a result later extended to semimetrics (Zadeh and Ghadiri 2015). He et al. propose a submodular objective that penalizes relevance with a pairwise similarity function. To satisfy the requirements for approximation guarantees, however, the relevance term easily overwhelms similarity (He et al. 2012). Abbassi et al. give a local-search 2-approximation for a maximization objective under matroid constraints (Abbassi et al. 2013). Küçüktunç et al. argue that some commonly used objectives can promote query-oblivious results, and propose a submodular function to overcome the issue (Küçüktunç et al. 2013). Ashkan et al. propose a polynomial-time solvable objective and an optimal greedy algorithm (Ashkan et al. 2015). Bhaskara et al. give an 8-approximation for a metric sum-min objective with matroid constraints (Bhaskara et al. 2016). The problem of diversification by minimization is closely related to the $k$ -Sparsest-Subgraph problem. Despite its inapproximability, special cases can be approximated or solved in polynomial time (Watrigant et al. 2012). Finally, Gollapudi and Sharma propose a set of desirable axioms for diversification objectives (Gollapudi and Sharma 2009), and show no objective can simultaneously satisfy all of them.

## Problem statement

*Notation* Lowercase boldface letters ($\mathbf {x}$) denote vectors, uppercase boldface letters denote matrices ($\mathbf {A}$), and uppercase light letters denote sets (*S*). We write $x_i$ to denote the *i*-th component of vector $\mathbf {x}$, and $\mathbf {A}_{ij}$ to denote the entry in the *i*-th row and *j*-th column of matrix $\mathbf {A}$. We denote by $\Vert \mathbf {x}\Vert$ the norm of vector $\mathbf {x}$. $\mathbb {P}\left[ {E}\right]$ is the probability of event *E* (the distribution will be clear from the context) and $\mathbb {E}\left[ {X}\right]$ is the expected value of a random variable *X*. Finally, ${\mathbb {R}}_+$ is the set of non-negative real numbers.

*Note* All proofs that are not given in the text can be found in the appendix.

We address a quite general form of sparse/diverse item selection, which we formulate as follows:

### Problem 1

Given a non-negative matrix $\mathbf {W}$ with zeros in the diagonal, find

$$\begin{aligned} \min \nolimits _{\mathbf {x} \in \{0,1\}^n} ~&\mathbf {x}^T\mathbf {W}\mathbf {x} + \mathbf {c}^T\mathbf {x}&\\ \text {subject to }\quad&\mathbf {1}^T\mathbf {x} = k .&\nonumber \end{aligned}$$

This problem formulation encompasses a wide variety of problems of interest, such as independent set or *k*-sparsest subgraph, and as such it is computationally hard. In fact, it can be shown that Problem 1 cannot be approximated within any multiplicative factor.

### Theorem 1

It is $\mathbf {NP}$-hard to approximate Problem 1 within any multiplicative factor.

In the next section we discuss the first step of our approach to obtain good solutions.

## Relaxation, rounding and analysis

We approach Problem 1 by tackling a natural relaxation, consisting simply of replacing the feasible set by $[0,1]^n$ to allow continuous solutions. We will first treat the problem of mapping one such continuous solution to a discrete one. Later we will treat the problem of obtaining good continuous solutions in cases of interest.

Assume we have obtained a solution $\mathbf {z} \in [0,1]^n$ to the “relaxed” variant of Problem 1. To construct a solution $\mathbf {x}\in \{0,1\}^n$ we adopt a randomized scheme: For each *i*, we set $x_i=1$ with probability $z_i$, and $x_i=0$ with probability $1-z_i$. We refer to this procedure as RandomizedRounding with input $\mathbf {z}$.

*Rounding with cardinality constraints* This form of randomized rounding is often easy to analyze in unconstrained settings, often leading to solutions with quality guarantees (Williamson and Shmoys 2011). However, its application to Problem 1 is not straightforward, because of the constraint $\mathbf {1}^T\mathbf {x}=k$, which may be violated if we round each coordinate *i* independently.

To address this issue, we propose a simple remedy: repeat the randomized rounding procedure *m* times, and pick a solution $\mathbf {x}$ that satisfies the constraint $\mathbf {1}^T\mathbf {x}=k$. There are two challenges with this approach: (*i*) show that a small number of trials *m* is sufficient to find a solution that satisfies the constraint, and (*ii*) analyze the quality of the solution, conditioned on feasibility. Note that even though the variables we *sample* are independent, the variables we *analyze* are not, due to conditioning. This makes the analysis significantly more difficult. We address these issues in the following two sections.

Note that there exist methods to randomly round vectors so as to obtain solutions respecting a cardinality constraint, but this entails introducing dependencies in the process, which has disadvantages. For instance, the method of Srinivasan (Srinivasan 2001) requires the construction of a binary tree, which limits parallelizability. The approach of Doerr (Doerr 2005) requires, for each entry of the vector, a number of coin flips in the order of the machine precision.

On the contrary, the simple rounding approach we take requires a single random experiment per vector entry, is fully parallelizable and trivial to implement. Moreover, our approach is easy to derandomize, using conditional expectations and the fact that Poisson-Binomial densities are efficiently computable using the Discrete Fourier Transform (Fernández and Williams 2010).

*Outline of the analysis* The rest of this section is devoted to the analysis of the proposed method, and is structured as follows:

- In Sect. 4.1 we show how to obtain a feasible solution with high probability.
- In Sect. 4.2 we analyze the expected performance of a feasible solution.
- Said analysis involves bounding ratios of Poisson-Binomial distributions, which is of independent interest and is carried out in Sect. 4.3
- In Sect. 4.4 we state the resulting approximation guarantees.

### Obtaining a feasible solution

The proposed randomized-rounding procedure does not guarantee a feasible solution: the resulting vector could have any number of entries equal to 1, but we require exactly *k*. Thus, *how many times do we need to run the* RandomizedRounding *procedure to obtain a feasible solution to Problem* 1*?*

Our analysis relies on the fact that the number of entries equal to 1 follows a Poisson-Binomial distribution, which we now define.

#### Definition 1

(Poisson-Binomial distribution) Let $X_1, \dots , X_n$ be independent, not necessarily identically distributed Bernoulli random variables. Then the variable $X=\sum _iX_i$ is said to follow a Poisson-Binomial distribution (Wang 1993).

If the success probability of the Bernoulli variable $X_i$ is $p_i$, then $\mathbb {E}\left[ {X}\right] =\sum _ip_i$. In our case, we are interested in the variable $\mathbf {x}^T\mathbf {x} = \sum _ix_i$. Note that $\mathbb {E}\left[ {\mathbf {x}^T \mathbf {x}}\right] =\sum _iz_i=k$.

We now analyze the number of attempts needed to guarantee a feasible solution with high probability.

#### Lemma 1

Let the set $S=\{\mathbf {x}_1, \dots , \mathbf {x}_m\}$ contain the output of *m* executions of RandomizedRounding with input vector $\mathbf {z}$, satisfying $\sum _iz_i=k$. Then for some $m = \mathcal {O} \left( \sqrt{k}\log \frac{1}{\delta } \right) ,$ and for sufficiently large *n* and *k*, with probability $1-\delta$ there exists at least one $\mathbf {x} \in S$ satisfying $\mathbf {x}^T\mathbf {x}=k$.

#### Proof

First, observe that since $\mathbb {E}\left[ {\mathbf {x}^T \mathbf {x}}\right] =k$, the probability of *k* successes is lower-bounded by the same event under a Binomial distribution with *n* tries and mean *k* (Hoeffding 1956), that is

$$\begin{aligned} \mathbb {P}\left[ {\mathbf {x}^T \mathbf {x}=k}\right] \ge {n \atopwithdelims ()k} \left( \frac{k}{n}\right) ^k \left( \frac{n-k}{n}\right) ^{n-k} = \frac{n!}{k!(n-k)!} \left( \frac{k}{n}\right) ^k \left( \frac{n-k}{n}\right) ^{n-k}. \end{aligned}$$

We use Stirling’s approximation of the factorial, $n!\approx \sqrt{2\pi n}\left( \frac{n}{e}\right) ^n$ to obtain, for sufficiently large *n* and *k*,

$$\begin{aligned} \mathbb {P}\left[ {\mathbf {x}^T \mathbf {x}=k}\right] \ge \frac{\sqrt{2\pi n}\left( \frac{n}{e}\right) ^n}{\sqrt{2\pi k}\left( \frac{k}{e}\right) ^k\sqrt{2\pi (n-k)}\left( \frac{n-k}{e}\right) ^{n-k}} \left( \frac{k}{n}\right) ^k \left( \frac{n-k}{n}\right) ^{n-k} \\=\frac{\sqrt{n}}{\sqrt{2\pi k(n-k)}} = \sqrt{\frac{n}{2\pi k(n-k)}} = \sqrt{\frac{1}{2\pi k(1-k/n)}} \ge \frac{1}{\sqrt{2\pi k}}. \end{aligned}$$

So we have $\mathbb {P}\left[ {\mathbf {x}^T \mathbf {x}\ne k}\right] \le 1-\frac{1}{\sqrt{2\pi k}} = \frac{\sqrt{2\pi k}-1}{\sqrt{2\pi k}}$. Now, observe that

$$\begin{aligned} \lim _{k\rightarrow \infty } \log \left( \frac{\sqrt{2\pi k}-1}{\sqrt{2\pi k}}\right) \approx \frac{1}{\sqrt{2\pi k}}, \end{aligned}$$

which follows from the fact that $\log \left( \frac{\sqrt{2\pi k}-1}{\sqrt{2\pi k}}\right) = -\left( \log (\sqrt{2\pi k}) - \log (\sqrt{2\pi k}-1)\right)$ is asymptotically equal to minus the derivative of $\log (\sqrt{2\pi k})$ w.r.t. $\sqrt{2\pi k}$. Thus, for sufficiently large *k*,

$$\begin{aligned} \mathbb {P}\left[ {\mathbf {x}^T \mathbf {x}\ne k}\right] \le \exp \left( -1/\sqrt{2\pi k}\right) . \end{aligned}$$

This implies that $\sqrt{2\pi k}\log \delta ^{-1}$ tries guarantee success with probability at least $1-\delta$, which concludes the proof. $\square$

### Analysis of feasible solutions

After obtaining a feasible solution using the rounding procedure, we analyze its quality with respect to the objective of Problem 1. First, observe the following:

1 $$\begin{aligned}&\mathbb {E}\left[ {\mathbf {x}^T\mathbf {W}\mathbf {x} +\mathbf {c}^T\mathbf {x}\bigm | \mathbf {x}^T\mathbf {x}=k}\right] = \sum _i\sum _j\mathbf {W}_{ij}\mathbb {P}\left[ {x_i=1, x_j=1 \bigm | \mathbf {x}^T\mathbf {x}=k}\right] \nonumber \\ {}&\qquad + \sum _i c_i \mathbb {P}\left[ {x_i=1 \bigm | \mathbf {x}^T\mathbf {x}=k}\right] \nonumber \\ {}&\quad = \sum _i\sum _{j\ne i}\mathbf {W}_{ij}\frac{\mathbb {P}\left[ {\mathbf {x}^T\mathbf {x}=k \bigm | x_i=1, x_j=1}\right] \mathbb {P}\left[ {x_i=1, x_j=1}\right] }{\mathbb {P}\left[ {\mathbf {x}^T\mathbf {x}=k}\right] } \nonumber \\ {}&\qquad +\sum _i c_i\frac{\mathbb {P}\left[ {\mathbf {x}^T\mathbf {x}=k \bigm | x_i=1}\right] \mathbb {P}\left[ {x_i=1}\right] }{\mathbb {P}\left[ {\mathbf {x}^T\mathbf {x}=k}\right] } \nonumber \\ {}&\quad = \!\sum _i\sum _{j\ne i}\mathbf {W}_{ij}z_iz_j\frac{\mathbb {P}\left[ {\mathbf {x}^T\mathbf {x}=k \bigm | x_i=1, x_j=1}\right] }{\mathbb {P}\left[ {\mathbf {x}^T\mathbf {x}=k}\right] } +\!\sum _ic_iz_i\frac{\mathbb {P}\left[ {\mathbf {x}^T\mathbf {x}=k \bigm | x_i=1}\right] }{\mathbb {P}\left[ {\mathbf {x}^T\mathbf {x}=k}\right] } . \end{aligned}$$

Here $\mathbf {z}$ is the obtained solution to the relaxed program. This equality shows that the expected performance of the rounded vector is within a factor of the quadratic-program solution, determined by a ratio of probabilities — rather, by the larger of two ratios. Next, we bound these ratios to derive our approximation guarantee.

To upper bound the probability ratios in Eq. (1), it will be more convenient to lower-bound their reciprocals:

$$\begin{aligned} \frac{\mathbb {P}\left[ {\mathbf {x}^T\mathbf {x}=k}\right] }{\mathbb {P}\left[ {\mathbf {x}^T\mathbf {x}=k \bigm | x_i=1, x_j=1}\right] } \,\text { and }\, \frac{\mathbb {P}\left[ {\mathbf {x}^T\mathbf {x}=k}\right] }{\mathbb {P}\left[ {\mathbf {x}^T\mathbf {x}=k \bigm | x_i=1}\right] }~, \end{aligned}$$

for all $i \ne j$. We will show in Theorem 3 that these reciprocals can be lower-bounded by a constant.

### Bounding ratios of Poisson binomial densities

We rely on a simple but crucial fact: the variable $(\mathbf {x}^T\mathbf {x} \mid x_i=1,x_j=1)$ is a shifted Poisson-Binomial with parameters $(z_1, \dots , z_{i-1}, z_{i+1}, \dots , z_{j-1}, z_{j+1}, \dots , z_n)$ (assuming $i<j$, without loss of generality).

Thus, we can define *Y* to be $(\mathbf {x}^T\mathbf {x} \mid x_i=1,x_j=1)-2$, or $\mathbf {x}^T\mathbf {x}$ conditioned by the event $x_i=1,x_j=1$, minus 2, and write

$$\begin{aligned}&\frac{\mathbb {P}\left[ {\mathbf {x}^T\mathbf {x}=k}\right] }{\mathbb {P}\left[ {\mathbf {x}^T\mathbf {x}=k \bigm | x_i=1, x_j=1}\right] } = \frac{\mathbb {P}\left[ {\mathbf {x}^T\mathbf {x}=k}\right] }{\mathbb {P}\left[ {Y=k-2}\right] } \\&\quad = z_iz_j + \frac{(z_i(1-z_j)+(1-z_i)z_j)\mathbb {P}\left[ {Y=k-1}\right] }{\mathbb {P}\left[ {Y=k-2}\right] } +\frac{(1-z_i)(1-z_j)\mathbb {P}\left[ {Y=k}\right] }{\mathbb {P}\left[ {Y=k-2}\right] }, \end{aligned}$$

Similarly, by defining $Z=(\mathbf {x}^T\mathbf {x} \mid x_i=1)-1$ we get

$$\begin{aligned} \frac{\mathbb {P}\left[ {\mathbf {x}^T\mathbf {x}=k}\right] }{\mathbb {P}\left[ {\mathbf {x}^T\mathbf {x}=k \bigm | x_i=1}\right] } = z_i + \frac{(1-z_i)\mathbb {P}\left[ {Z=k}\right] }{\mathbb {P}\left[ {Z=k-1}\right] }. \end{aligned}$$

The main challenge in bounding the above expressions lies in the fractions of probability densities, such as $\frac{\mathbb {P}\left[ {Y=k-1}\right] }{\mathbb {P}\left[ {Y=k-2}\right] }$. We now derive general bounds for fractions of this kind, which are of independent interest and will constitute the cornerstone of our approximation results.

#### Theorem 2

Let *Y* be a Poisson-Binomial variable with expected value $\mathbb {E}\left[ {Y}\right] =c-\alpha$, for some $c \in {\mathbb {N}}$ and $0 \le \alpha < 1$. Then

$$\begin{aligned} \frac{\mathbb {P}\left[ {Y=c}\right] }{\mathbb {P}\left[ {Y=c-1}\right] } \ge 1-\alpha . \end{aligned}$$

The full proof is given in the appendix, but we provide an outline here. Analyzing this ratio is challenging given the form of the Poisson-Binomial density, which involves the summation of an exponential number of terms. To sidestep this inconvenience we resort to two tools. First, we characterize the ratio for distributions satisfying the Karush-Kuhn-Tucker conditions (Boyd et al. 2004). Next, we combine said characterization with the symmetry of the Poisson-Binomial density to reveal that optimal points have a tractable analytical form, which in turn leads to the bound.

We can generalize the previous bound by relying on the log-concavity of the Poisson-Binomial density (Hillion et al. 2017). The log-concavity implies that $\mathbb {P}\left[ {Y=x}\right] ^2 \ge \mathbb {P}\left[ {Y=x-1}\right] \mathbb {P}\left[ {Y=x+1}\right]$, for all valid choices of *x*. From this it is easy to obtain the following

#### Corollary 1

Let *Y* be a Poisson-Binomial variable with expected value $\mathbb {E}\left[ {Y}\right] =c-\alpha$, for some $c \in {\mathbb {N}}$ and $0 \le \alpha < 1$. For any $1\le i < c)$,

$$\begin{aligned} \frac{\mathbb {P}\left[ {Y=c}\right] }{\mathbb {P}\left[ {Y=c-i}\right] } \ge (1-\alpha )^i. \end{aligned}$$

Finally, relying on these results we can show that the ratios arising in Eq. (1), which relates our solutions to the optimum, are bounded from above by a constant.

#### Theorem 3

Let $X=\sum _i X_i$ be a Poisson-Binomial variable with expectation $\mathbb {E}\left[ {X}\right] =k$, for some $k\in {\mathbb {N}}$.

$$\begin{aligned} \frac{{\mathbb {P}\left[ {X=k \bigm | X_i=1, X_j=1}\right] }}{\mathbb {P}\left[ {X=k}\right] } \le 1.73 ~\text { and }~ \frac{{\mathbb {P}\left[ {X=k \bigm | X_i=1}\right] }}{\mathbb {P}\left[ {X=k}\right] } \le \frac{4}{3}. \end{aligned}$$

### Approximation guarantees

Combining Eq. (1) with the above results immediately yields our main results regarding the quality of independent randomized rounding with cardinality constraints.

#### Theorem 4

Consider an instance of Problem 1 with input $\mathbf {W}$, $\mathbf {c}$ and a vector $\mathbf {z} \in [0,1]^n$ satisfying $\sum _i z_i=k$. Let $\mathbf {x}$ be a solution obtained by running RandomizedRounding with input $\mathbf {z}$. Then

$$\begin{aligned} \mathbb {E}\left[ {\mathbf {x}^T\mathbf {W} \mathbf {x} + \mathbf {c}^T\mathbf {x}\bigm | \mathbf {x}^T\mathbf {x}=k}\right] \le 1.73(\mathbf {z}^T \mathbf {W} \mathbf {z} + \mathbf {c}^T\mathbf {z}). \end{aligned}$$

The expected performance of our rounding scheme is thus within a constant of the relaxed solution. Armed with this fact we employ Markov’s inequality to show the next result.

#### Theorem 5

Consider an instance of Problem 1, and a vector $\mathbf {z} \in [0,1]^n$. There exists a randomized algorithm that runs in

$$\begin{aligned}\mathcal {O} \left( T_{QF}\frac{\sqrt{k}\log ^2(\delta ^{-1})}{\epsilon }\right) \end{aligned}$$

( where $T_{QF}$ is the time required to compute a quadratic form of size *n*) and outputs a solution $\mathbf {x}$ satisfying, with probability $1-\delta$,

$$\begin{aligned} \mathbf {x}^T \mathbf {W} \mathbf {x} + \mathbf {c}^T\mathbf {x} \le 1.73(1+\epsilon )(\mathbf {z}^T \mathbf {W} \mathbf {z} + \mathbf {c}^T\mathbf {z}). \end{aligned}$$

## Finding continuous solutions

We have shown that independent rounding allows us to transform a continuous solution to a quadratic programming problem into a discrete with loss bounded by a small constant. Of course the quality of the solution will ultimately depend on the quality of the continuous solution to the relaxed program. As the matrix we consider is indefinite, finding a good continuous solution might itself be challenging. However, in some cases it is possible to do this efficiently. In the next section we will analyze one such case.

*Convexification* The matrix $\mathbf {W}$ can be easily modified so that it becomes convex, by shifting the diagonal. This introduces an additive term of at most $k|\lambda _n|$, where $\lambda _n$ is the smallest eigenvalue of matrix $\mathbf {W}$. Thus, if the negative part of the spectrum is bounded, this term can be negligible, or overwhelmed by the quadratic term. An example is shown in Sect. 6. Other approaches might be used in some cases to obtain better additive terms. For instance, one can trade off the magnitude of the shift and running time, as a small number of negative eigenvalues — as explained next — can result in manageable running times for moderately sized data.

*Low-rank matrices* An indefinite quadratic program can be approximated in time exponential in the number of negative eigenvalues of $\mathbf {W}$ (Vavasis 1992). Thus, if the rank of $\mathbf {W}$ is bounded by a constant, a near-optimal continuous solution — and therefore one to the 0-1 program — can be obtained in polynomial time. Low-rank approximations can be used to ensure this is the case.

## Application to search result diversification

Here we show that we can use our previous results to obtain a provably good solution in a special case of our problem.

We consider a set ${{\mathcal {W}}}=\{{\mathbf {w}}_1,\ldots ,{\mathbf {w}}_n\}$ of *n* items, each represented as a vector in ${\mathbb {R}}^d$, and a similarity function $\sigma$ which maps pairs of items of ${\mathcal {W}}$ into the interval [0, 1], i.e., $\sigma : {\mathcal {W}}\times {\mathcal {W}}\rightarrow [0,1]$. The larger the value $\sigma (\mathbf {w}_i,\mathbf {w}_j)$, the more similar the items ${\mathbf {w}_i}$ and ${\mathbf {w}_j}$. We assume that $\sigma$ satisfies two properties:

(id): $\sigma (\mathbf {w},\mathbf {w})=1$, for any $\mathbf {w} \in {\mathbb {R}}^d$; and
(psd): for any subset $\{\mathbf {w}_1, \dots , \mathbf {x}_n\}\subseteq {\mathbb {R}}^d$, the matrix $\left( \sigma (\mathbf {w}_i,\mathbf {w}_j)\right) _{i,j=1}^n$ is symmetric positive semidefinite.

Note that these properties (id) and (psd) are satisfied by a wide range of commonly-used functions, such as Jaccard similarity, rbf kernels, or cosine similarity of non-negative data.

The goal is, given a query and a natural number *k*, to return a set ${\mathcal {S}}$ of *k* items of ${\mathcal {W}}$ (i.e., ${\mathcal {S}}\subseteq {\mathcal {W}}$ and $|{\mathcal {S}}|=k$) that are relevant but not too similar to each other. Further, we use a *relevance loss* function $\rho (\mathbf {w})$, which is a measure of the cost incurred by adding item $\mathbf {w}$ into ${\mathcal {S}}$. The smaller the value of this loss function for an item, the more relevant it is considered to be.

### Problem 2

(MinSumSim) Consider a set ${{\mathcal {W}}}=\{{\mathbf {w}}_1,\ldots ,{\mathbf {w}}_n\}$ of size *n*, an integer *k*, and $\lambda \in {\mathbb {R}}_+$. Our goal is to choose a subset ${\mathcal {S}}$ of *k* items from ${\mathcal {W}}$ to minimizewhere $\rho$ is a *relevance loss* function and $\sigma : {\mathcal {W}}\times {\mathcal {W}}\rightarrow [0,1]$ is a *similarity* function satisfying the properties (id) and (psd).

We define an $n\times n$ matrix $\mathbf {W}$ as

$$\begin{aligned} \mathbf {W}_{ij}= {\left\{ \begin{array}{ll} \sigma (\mathbf {w}_i,\mathbf {w}_j) &{} \text{ if } i\ne j \\ 0 &{} \text{ otherwise }, \end{array}\right. } \end{aligned}$$

and a vector $\mathbf {c}$ such that $c_i=\lambda \rho (\mathbf {w}_i)$. It is clear that MinSumSim is an instance of Problem 1.

In order to efficiently find a continuous solution to the relaxed problem, we inflate $\mathbf {W}$. We will show that in doing so we introduce a bounded additive term in the approximation error.

In particular, define $\mathbf {W}'=\mathbf {W}+\mathbf {I}$. Note that $\mathbf {W}'$ is the similarity matrix from problem MinSumSim, and is thus positive semidefinite. Let $\mathbf {y}_{*}$ and $\mathbf {x}_{*}$ be optimal solutions of the MinSumSim problem with matrices $\mathbf {W}'$ and $\mathbf {W}$, respectively. Then

$$\begin{aligned} \mathbf {y}_{*}^{T}\mathbf {W}'\mathbf {y}_{*} + \mathbf {c}^T\mathbf {y}_{*} \le&\quad \mathbf {x}_{*}^{T}\mathbf {W}'\mathbf {x}_{*} + \mathbf {c}^T\mathbf {x}_{*}&\text {(optimality of }\mathbf {y}_{*}\text {)} \\ =&\quad \mathbf {x}_{*}^{T}\mathbf {W}\mathbf {x}_{*} + \mathbf {c}^T\mathbf {x}_{*} + \sum _{i}x_i^2&\text {(definition of }\mathbf {W}'\text {)}. \end{aligned}$$

Since $\sum _{i}x_i^2\le k$ (note that the sum constraint is binding), the following result follows.

### Lemma 2

Let $\mathbf {y}_{*}$ and $\mathbf {x}_{*}$ be optimal solutions of the MinSumSim problem with matrices $\mathbf {W}'$ and $\mathbf {W}$ respectively, Then

$$\begin{aligned} \mathbf {y}_{*}^{T}\mathbf {W}\mathbf {y}_{*} + \mathbf {c}^T\mathbf {y}_{*} \le \mathbf {x}_{*}^{T}\mathbf {W}\mathbf {x}_{*} + \mathbf {c}^T\mathbf {x}_{*} + k. \end{aligned}$$

Since convex quadratic programs with linear constraints are efficiently solvable, we can obtain an optimal continuous solution to the problem using $\mathbf {W}'$ in the quadratic term. Applying Theorem 5, we immediately obtain the following result.

### Theorem 6

Consider an instance $({\mathcal {W}},k,\lambda )$ of the MinSumSim problem, with $\lambda \ge 1$ and $|{\mathcal {W}}|=n$. Let $OPT$ by the value of the optimal solution. Then, there exists a randomized algorithm that runs in time $\mathcal {O} \left( T_{QP} + T_{QF}\frac{\sqrt{k}\log ^2(\delta ^{-1})}{\epsilon }\right)$ (where $T_{QP}$ is the time required to solve a convex quadratic program of size $n\times n$ with linear constraints and $T_{QF}$ is the time required to compute a quadratic form of size *n*) and outputs a solution set ${\mathcal {S}}\subseteq {\mathcal {W}}$ satisfying, with probability $1-\delta$,

$$\begin{aligned} cost ({\mathcal {S}}) \le 1.73(1+\epsilon )( OPT +k). \end{aligned}$$

*Scalability and complexity analysis* The quadratic program can be solved using interior-point algorithms in roughly $\mathcal {O} (n^3)$ time (Potra and Wright 2000). Available solvers are heavily optimized, and in practice are much faster than what worst-case complexity suggests. This is further evidenced in our experimental evaluation. The randomized rounding can be trivally parallelized, as the samples are independent of each other.

*Practical implementation* It should be noted that it is not in general necessary to construct the pairwise-similarity matrix in order to solve the problem. More details are given in the appendix.

*Scaling to large datasets* Our experiments demonstrate how our algorithm can process large datasets in reasonable amounts of time. However, in time-sensitive scenarios one might choose to trade off quality for running time. We propose a method to accomplish this. Given an instance $({\mathcal {W}}, k, \lambda )$ of size *n* we sample a small number of items (say $\sqrt{n}$) from ${\mathcal {W}}$, uniformly without replacement, to produce a new instance $({\mathcal {{\tilde{W}}}}, k, \lambda )$, which can be solved much faster. We sample and solve a large number of such instances, which can be done in a distributed fashion, and take the union of the solutions, each of size *k*, to produce a final instance $({\mathcal {{\hat{W}}}}, k, \lambda )$, which we solve to produce the final output. We show in the experiments how this scheme can provide significant speedup at a small quality cost.

## Experimental evaluation

We test our algorithm on real-world document collections, and compare its performance against two greedy algorithms from the literature. Our experimental evaluation shows how our method consistently outperforms competitors.

*Datasets* We select publicly available collections of documents resulting from queries. Since each document is query-dependent, and the same document could be returned by multiple queries (with some varying features), we consider query-document pair to be unique. We consider datasets from the LETOR (Qin and Liu 2013; Qin et al. 2010), Istella (Istella extended learning to rank) (Lucchese et al. 2018) and UCI (Dua and Graff 2017) collections. Their characteristics are summarized in Table 1, and a more detailed description is given in the supplement.

**Table 1:** Collections of query-related documents used in the experiments: number of query-document pairs(*N*); dimensionality(*d*); range of relevance score $r_i$

Dataset	*N*	*d*	$r_i$	Data type
MQ2007	69 623	46	{0,1,2}	TF-IDF, BM25, LMIR
MQ2008	15 211	46	{0,1,2}	TF-IDF, BM25, LMIR
Gov2003hp	147 606	64	{0,1,2}	TF-IDF, BM25, LMIR
Census	2 458 285	68		
Istella	5 308 608	220	{0,1,2,3,4}	BM25

*Baselines* We consider two greedy methods from the literature: Edge-Greedy, (Gollapudi and Sharma 2009) and Node-Greedy, (Borodin et al. 2017). Both give 2-approximations for maximization formulations. We run Node-Greedy using 10 and 50 tries, as the first item is chosen at random. We refer to our algorithm as MinSumSim.

These algorithms are straightforward to adapt to our minimization setting. We also considered a local-search heuristic, as proposed by (Borodin et al. 2017). However, running times became unmanageable and improvements, as observed by (Borodin et al. 2017), were negligible. It should be noted that existing methods for diversification either tackle very different objectives to ours and are not comparable, or address similar objectives using greedy heuristics. Thus, we believe that the baselines we consider cover a good part of the available methods for an objective like ours.

In all experiments we use cosine similarity. We normalize relevances document-query pairs in the given datasets to be in (0, 1]. If the relevance of a given document-query pair is *r*, we set its relevance-loss score to be $\rho (\mathbf {x})=1+\log (1/r)$. All our experiments are run on a Linux node with 14-core CPUs and 128 GB of RAM. We use the MOSEK fusion API as our quadratic program solver on python 3.7.6. The source code is publicly available.^1^

**Fig. 1:**
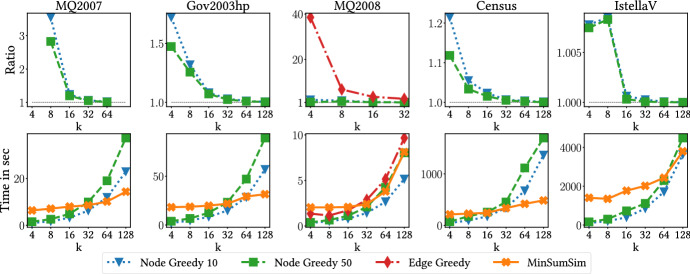
Comparison of the performance of baselines compared to our method (MinSumSim). Top: relative performance, measured by the ratio cost of baseline versus the cost of our method. Bottom: running times. Lower is better in both cases

**Fig. 2:**
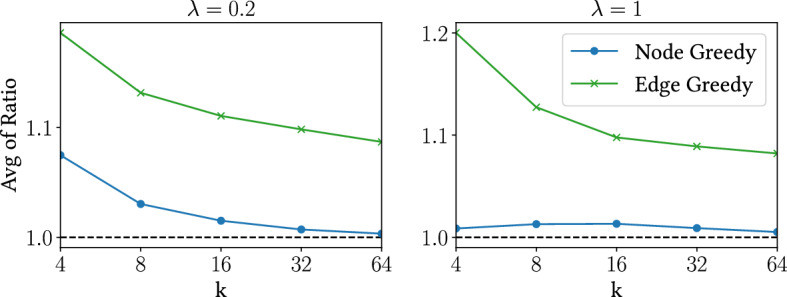
Performance for different values of $\lambda$

*Performance evaluation* We first run the algorithms to find the most dissimilar items on the complete datasets, that is, the case $\lambda =0$. We restrict Edge-Greedy to the smallest one due to scalability limitations. The results are shown in Fig. 1 (top). We report the ratio of the solution returned by the competing methods to ours, that is, if e.g., Node-Greedy achieves a solution of cost *G* and MinSumSim a solution of cost *A*, we report the ratio *G*/*A*. Note how our method consistently achieves superior performance, by a large factor for small *k*. Running times are reported in Fig. 1 (bottom). Our method, though initially slightly slower than the greedy alternatives, is much less sensitive to the value of *k*.

*The effect of parameter* $\lambda$ We use the document-query pairs in the datasets, along with their corresponding relevance-loss scores, to evaluate how our method compares to the greedy algorithms when $\lambda$ increases. We extract 100 queries from Istella, comprised of 5 000 documents each, and run the algorithms for $\lambda =0.2,1$, taking the average of performance ratios. The results are shown in Fig. 2. When $\lambda$ is large, Node-Greedy is expected to do better. We observe this to be the case in our experiments, where its performance seems to converge with that of our method. Edge-Greedy remains ineffective.

**Fig. 3:**
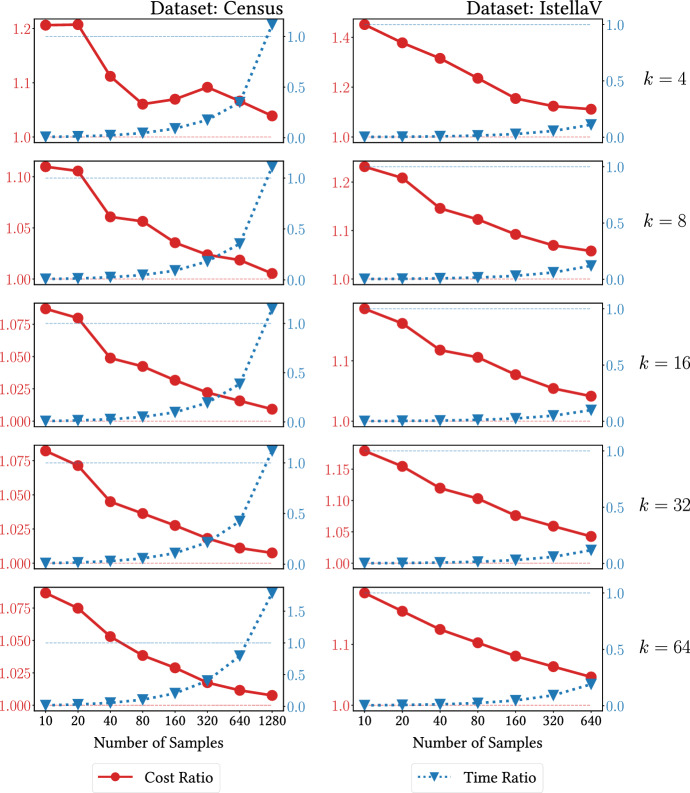
Performance for the randomized subsampling scheme

*Subsampling* Here we illustrate the effects of the scheme described in Sect. 6, designed to obtain approximate solutions at a reduced computational cost. Our goal is to assess how the number of random samples impacts the quality of the solution, as well as the running time. We run our algorithm on the two larger datasets (Census and Istella), taking an increasing number of random samples from the dataset. We repeat the experiment for increasing values of *k*. Figure 3 shows the results. We simultaneously report the ratio of the cost of the obtained solution to the solution obtained by solving the complete instance (lower is better) and the speedup. The benefits of this approach are of course more noticeable as the size of the dataset increases. Note how on Istella, the larger of the two datasets, we can achieve $10\times$ speed-up and obtain a solution within approximately 5% of the one achieved by solving the full instance. The amount of speedup gained clearly depends on the capabilities of the distributed environment and the quality of the implementation.

*Greed is not always good* To better understand the weaknesses of greedy methods, we design an example where their performance is poor.

We now describe the proposed structure. Consider a set *X* of *n* documents. We consider a topic model comprised of *d* topics. For each item $\mathbf {x} \in X$, $x_i \in {\mathbb {R}}_+$ indicates how relevant a topic *i* is to $\mathbf {x}$. We assume that *X* is partitioned as $X= Y\cup Z$. Documents in *Y* are mostly uncorrelated with each other, as are documents in *Z*. However, some documents in *Y* correlate with some documents in *Z*. The setting can be achieved with a matrix with the following structure, where $a\gg b$ (we highlight *a* in boldface to emphasize the structure):

$$\begin{aligned} \mathbf {A}=\left( \begin{array}{cccccccccc} \mathbf {a}&{}\quad b&{}\quad b&{}\quad \mathbf {a}&{}\quad b&{}\quad b&{}\quad \mathbf {a}&{}\quad b&{}\quad b&{}\quad \\ b&{}\quad \mathbf {a}&{}\quad b&{}\quad b&{}\quad \mathbf {a}&{}\quad b&{}\quad b&{}\quad \mathbf {a}&{}\quad b&{}\quad \\ b&{}\quad b&{}\quad \mathbf {a}&{}\quad b&{}\quad b&{}\quad \mathbf {a}&{}\quad b&{}\quad b&{}\quad \mathbf {a}&{}\quad \cdots \\ \mathbf {a}&{}\quad b&{}\quad b&{}\quad \mathbf {a}&{}\quad b&{}\quad b&{}\quad \mathbf {a}&{}\quad b&{}\quad b&{}\quad \\ b&{}\quad \mathbf {a}&{}\quad b&{}\quad b&{}\quad \mathbf {a}&{}\quad b&{}\quad b&{}\quad \mathbf {a}&{}\quad b&{}\quad \\ b&{}\quad b&{}\quad \mathbf {a}&{}\quad b&{}\quad b&{}\quad \mathbf {a}&{}\quad b&{}\quad b&{}\quad \mathbf {a}&{}\quad \cdots \\ \mathbf {a}&{}\quad \mathbf {a}&{}\quad \mathbf {a}&{}\quad b&{}\quad b&{}\quad b&{}\quad b&{}\quad b&{}\quad b&{}\quad \\ b&{}\quad b&{}\quad b&{}\quad \mathbf {a}&{}\quad \mathbf {a}&{}\quad \mathbf {a}&{}\quad b&{}\quad b&{}\quad b&{}\quad \\ b&{}\quad b&{}\quad b&{}\quad b&{}\quad b&{}\quad b&{}\quad \mathbf {a}&{}\quad \mathbf {a}&{}\quad \mathbf {a}&{}\quad \\ &{}\quad &{}\quad &{}\quad &{}\quad &{}\quad \vdots &{}\quad &{}\quad &{}\quad &{} \end{array} \right) . \end{aligned}$$

Notice that each triplet of rows consist of rows that are completely uncorrelated with each other, but correlated with rows from other triplets.

To consider a more realistic scenario, we build a generative model based on the above structure: entries in positions with $\mathbf {a}$ follow a gamma distribution with shape parameter $\alpha$, and entries in positions labeled $b$ follow a gamma distribution with parameter $\beta$, with $\alpha \gg \beta$. These matrices simulate random realizations of topic-model vectors, correlating strongly with some topics and weakly with others.

Although the given structure may appear artificial, we now describe an example of a use case that could give rise to a similar structure.

We consider the topics in the corpus to correspond to themes usually covered in news articles, such as politics, sports, entertainment, etc. Furthermore, these topics are further broken down into subtopics. In the example matrix above, each column triplet corresponds to a topic (e.g. politics) and each column is a subtopic (e.g. domestic, international, etc.). The first six rows are set *Y*, which contains articles summarizing a selection of key events in various topics (e.g. a daily summary of headlines). The rest of the rows, *Z*, contain articles focused broadly on one topic, covering various subtopics. As the articles in *Y* pick a random selection of a few of the corresponding subtopics, they are weakly correlated with each other. However, they are fairly correlated with the more focused articles from |*Z*|.

Absent relevance scores, a greedy algorithm might start selecting an article from *Y*, and will eventually be forced to pick strongly correlated articles from *Y* or *Z*. On the contrary, our QP formulation makes a choice based on the global structure of the matrix, and will only choose articles from *Z*, resulting in a less correlated selection.

Of course, the previous example is idealized. However, for the greedy methods to fail it is not necessary for the data set to be exactly like this. It suffices that the data contains a similar structure, and the initial state of the algorithm to be a document in *Y*.

**Fig. 4:**
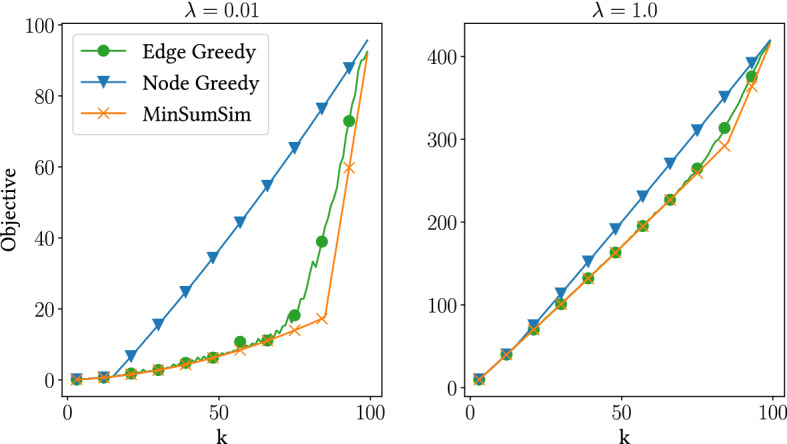
Performance of algorithms on adversarial example

To make our case stronger, we run the algorithms on such an adversarial example. Figure 4 shows the results. When the relevance term is small, greedy methods can fail to yield acceptable solutions. Edge-Greedy, as expected, is more robust than Node-Greedy in this case, as it inspects pairwise correlations for the initial choice, at an increased computational cost.

### Independent versus dependent rounding

**Fig. 5:**
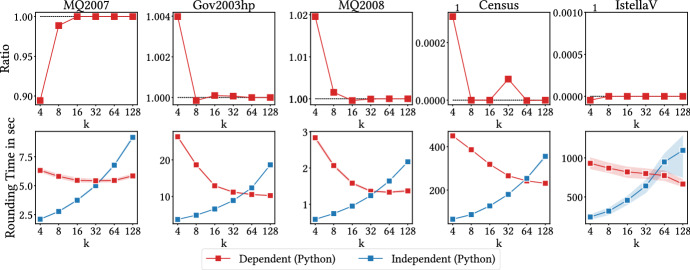
Comparison of the performance of the independent randomized rounding algorithm to the dependent randomized rounding algorithm for the MinSumSim problem implemented in python. Top: relative performance, measured by the ratio cost of baseline vs. the cost of our method. Bottom: running times of the randomized rounding algorithms

We compare the performance of the independent randomized rounding algorithm proposed in Sect. 4 to the dependent scheme proposed by Srinivasan (Srinivasan 2001). We repeat the previous experiment, now considering both rounding methods. We again report the cost ratios, but we only report the running times pertaining to the rounding algorithms, as solving the quadratic program is independent of the rounding technique employed. The results are shown in Fig. 5. Note how the cost ratio (top) is always close to 1. The differences seen at small values of *k* do not consistently favour any of the algorithms. We note here that in order to reliably obtain good values of the objective using Srinivasan’s method, it was necessary to shuffle the fractional vectors at each rounding attempt. This is presumably due to the low variance of the method, and the fact that we optimize a proxy of our objective function, with additive error.

The running times of the rounding algorithm are reported in Fig. 5 (bottom). The proposed independent algorithm is faster at small *k*. However, when *k* grows significantly it becomes slower as a consequence of the ${\varOmega } (\sqrt{k})$ tries required to obtain a feasible solution with theoretical guarantees (Lemma 1). Note that this dependency can trivially be mitigated with further parallelization. At first it might seem striking that the dependent algorithm exhibits decreasing running times as *k* increases. Upon further analysis, we find that this trend is caused by a decreasing percentage of fractional variables in *z*, the solution to the relaxed quadratic program. A higher percentage of fractional variables corresponds to a taller binary tree in the dependent algorithm, which in turn results in higher running times. We emphasize that our approach can also take benefit of this condition. The entries equal to 0 can be discarded, and any entries equal to 1 effectively reduce the value of *k*, which in turn reduces the number of required attempts. For simplicity, we did not implement these optimizations, and thus the reported running times may be slightly pessimistic for our method.

**Fig. 6:**
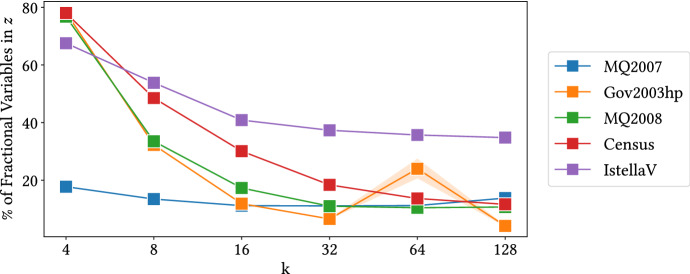
Percentage of fractional variables in $\mathbf {z}$ as a function of *k* for different datasets

To provide further insight on this point, we report the percentage of fractional variables in *z* for all the datasets in Fig. 6. Note how the number decreases as *k* increases, which explains the observed trend in running times for the dependent algorithm.

Finally, note how the impact of the running time of either optimized randomized rounding algorithm is insignificant compared to the time to solve a quadratic problem ($T_{QP}$) and the time to compute a quadratic form of size *n* ($T_{QF}$) for the MinSumSim problem. Nevertheless, we remind the reader that the proposed method can be used in conjunction with any other algorithm that requires rounding. The performance gains for small values of *k* can be significant in other contexts.

*Parallelizability.* One of the main advantages of the proposed rounding method, other than its simplicity, is its parallelizability. Even though we need to produce a number of rounded vectors in order to obtain quality guarantees, each of these vectors can be created in full parallel fashion. Further, each of the entries can be rounded independently (thus in parallel) as well. This is in contrast to the dependent method, which requires ${\varOmega } (\log n)$ sequential steps due to the necessary variable pairing. We thus attempt to obtain an estimate of how our method would fare when equipped with perfect parallelization, in comparison to the dependent method.

We measure the time it takes to obtain a single rounded solution, using synthetic fractional vectors of increasing size, with both our independent method and Srinivasan’s dependent method. In order to generate Poisson-Binomial distributions with user-defined mean and concentration, we consider the terms of a geometric series summing up to *k*. That is, the probability that the *i*-th experiment is 1 is $\mathbb {P}\left[ {X_i=1}\right] = ar^i$, where $a=\frac{k(1-r)}{1-r^n}$ and *r* is set to control concetration (the smaller *r*, the more concentrated the distribution). Using the formula for the geometric series, it is easy to check that

$$\begin{aligned} \sum _{i=0}^{n-1}ar^i = k. \end{aligned}$$

Further, if *r* satisfies $r-r^n/k \ge (k-1)/k$, all entries will be between 0 and 1.

**Fig. 7:**
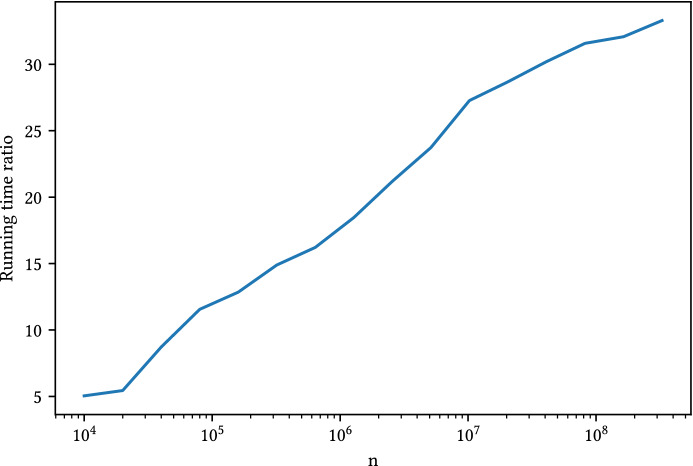
Comparison of the time taken by each of the rounding methods to generate a single solution

We generate vectors of size $n\in [10^4, 10^9]$, with $k=2$ and $r=1-10^{-6}$. The results, shown in Fig. 7, illustrate how our method can be up to 30 times faster when rounding large vectors. Of course, these speed-up factors will be mitigated in real scenarios due to the overhead necessary to run the methods in parallel.

## Conclusions

In this paper we have considered the problem of search-result diversification for similarity-type measures. We have proposed a formulation of the problem of diversification in minimization form, and proposed an efficient, scalable algorithm with approximation guarantees. Our experimental results show how our method consistently outperforms alternatives from the literature, while being comparable in speed and sometimes faster. Our approach is a significant step towards bridging a gap between theory and practice, as similarity functions are widely used by practitioners, while the theory of diverse-item selection relies almost exclusively in distance functions.

As future work we would like to determine whether our approximation guarantees can be improved. In particular, it would be interesting to explore natural alternative minimization objectives for diversification, which admit multiplicative approximation guarantees.

## Figures and Tables

**Fig. 8:**
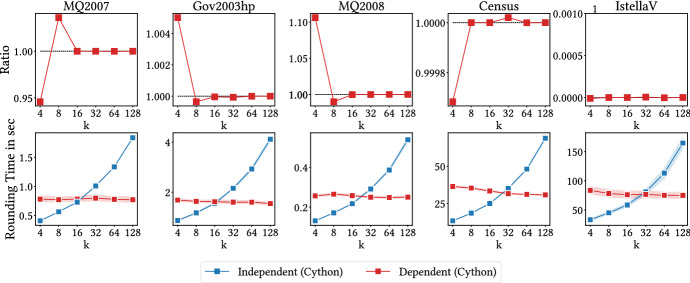
Comparison of the performance of the independent randomized rounding algorithm to the dependent randomized rounding algorithm implemented in C**++**. Top: relative performance, measured by the ratio cost of baseline vs. the cost of our method. Bottom: running times of the randomized rounding algorithms

## Appendix

### Proof of theorem 1

Consider an undirected, simple, loop-free graph with adjacency matrix $\mathbf {A}$, and an instance of MinSumSim where $\lambda =0$, and $\sigma (\mathbf {x}_i,\mathbf {x}_j) = \delta \mathbf {A}_{ij}$, for some $\delta \in {\mathbb {R}}_+$. By making $\delta$ small enough, we can ensure that property (psd) of $\sigma$ is satisfied. If there exists an independent set of size *k*, a multiplicative approximation of MinSumSim would reveal it. Thus, running the algorithm for all possible values of *k* would allow us to find the largest independent set in the graph. $\square$

The following technical lemma is a key ingredient in the proof of the bounds.

### Lemma 3

If $$ \nabla _{p_i}\frac{\mathbb {P}\left[ {Y_{\mathbf {p}}=k}\right] }{\mathbb {P}\left[ {Y_{\mathbf {p}}=k-1}\right] } = \nabla _{p_j}\frac{\mathbb {P}\left[ {Y_{\mathbf {p}}=k}\right] }{\mathbb {P}\left[ {Y_{\mathbf {p}}=k-1}\right] }$$ then $$p_i=p_j$$.

### Proof

We first expand the gradient. We multiply it by its denominator, which is constant with respect to the choice of *i*, for ease of presentation.

$$\begin{aligned}&\mathbb {P}\left[ {Y_{\mathbf {p}}=k-1}\right] ^2\nabla _{p_i}\frac{\mathbb {P}\left[ {Y_{\mathbf {p}}=k}\right] }{\mathbb {P}\left[ {Y_{\mathbf {p}}=k-1}\right] } \\&\quad = \nabla _{p_i}\left( \mathbb {P}\left[ {Y_{\mathbf {p}}=k}\right] \right) \mathbb {P}\left[ {Y_{\mathbf {p}}=k-1}\right] - \nabla _{p_i}\left( \mathbb {P}\left[ {Y_{\mathbf {p}}=k-1}\right] \right) \mathbb {P}\left[ {Y_{\mathbf {p}}=k}\right] \\&\quad = \left( \mathbb {P}\left[ {Y_{\mathbf {p}_{-i}}=k-1}\right] -\mathbb {P}\left[ {Y_{\mathbf {p}_{-i}}=k}\right] \right) \mathbb {P}\left[ {Y_{\mathbf {p}}=k-1}\right] \\&\qquad - \left( \mathbb {P}\left[ {Y_{\mathbf {p}_{-i}}=k-2}\right] -\mathbb {P}\left[ {Y_{\mathbf {p}_{-i}}=k-1}\right] \right) \mathbb {P}\left[ {Y_{\mathbf {p}}=k}\right] \\&\quad = \left( p_j\mathbb {P}\left[ {Y_{\mathbf {p}_{-i,j}}=k-2}\right] +(1-p_j)\mathbb {P}\left[ {Y_{\mathbf {p}_{-i,j}}=k-1}\right] \right) \mathbb {P}\left[ {Y_{\mathbf {p}}=k-1}\right] \\&\qquad - \left( p_j\mathbb {P}\left[ {Y_{\mathbf {p}_{-i,j}}=k-1}\right] +(1-p_j)\mathbb {P}\left[ {Y_{\mathbf {p}_{-i,j}}=k}\right] \right) \mathbb {P}\left[ {Y_{\mathbf {p}}=k-1}\right] \\&\qquad - \left( p_j\mathbb {P}\left[ {Y_{\mathbf {p}_{-i,j}}=k-3}\right] +(1-p_j)\mathbb {P}\left[ {Y_{\mathbf {p}_{-i,j}}=k-2}\right] \right) \mathbb {P}\left[ {Y_{\mathbf {p}}=k}\right] \\&\qquad - \left( p_j\mathbb {P}\left[ {Y_{\mathbf {p}_{-i,j}}=k-2}\right] +(1-p_j)\mathbb {P}\left[ {Y_{\mathbf {p}_{-i,j}}=k-1}\right] \right) \mathbb {P}\left[ {Y_{\mathbf {p}}=k}\right] . \end{aligned}$$

Since $\mathbb {P}\left[ {Y_{\mathbf {p}}=c}\right]$ and $\mathbb {P}\left[ {Y_{\mathbf {p}_{-i,j}}=c}\right]$ do not vary if we switch *i* and *j*, for any $\mathbf {p}$ we can pick constants *a*, *b* such that

$$\begin{aligned}&\nabla _{p_i}\frac{\mathbb {P}\left[ {Y_{\mathbf {p}}=k}\right] }{\mathbb {P}\left[ {Y_{\mathbf {p}}=k-1}\right] } = ap_j+b \text { and} \\&\nabla _{p_j}\frac{\mathbb {P}\left[ {Y_{\mathbf {p}}=k}\right] }{\mathbb {P}\left[ {Y_{\mathbf {p}}=k-1}\right] } = ap_i+b, \end{aligned}$$

which proves the lemma. $\square$

### Proof of Theorem 2

We consider the variable *Y* to be parametrized by $\mathbf {p} = (p_1, \dots , p_n)^T$. In order to provide a lower bound, we use Karush-Kuhn-Tucker (KKT) theory to characterize solutions of the following optimization problem with linear —and thus qualified— constraints.

$$\begin{aligned} \min _{\mathbf {p}} \quad&\frac{\mathbb {P}\left[ {Y=c}\right] }{\mathbb {P}\left[ {Y=c-1}\right] } \\ \text{ s.t. } \quad&0 \le p_i \le 1 \quad&i=1,\dots , n \\ \quad&\sum _ip_i = c-\alpha \end{aligned}$$

We rewrite the problem in canonical minimization form:

$$\begin{aligned} \min _{\mathbf {p}} \quad&\frac{\mathbb {P}\left[ {Y=c}\right] }{\mathbb {P}\left[ {Y=c-1}\right] } \\ \text{ s.t. } \quad&-p_i \le 0 \quad&i=1,\dots , n \\ \quad&p_i -1\le 0 \quad&i=1,\dots , n \\ \quad&\sum _ip_i -c + \alpha = 0 \end{aligned}$$

We introduce KKT multipliers $\alpha _i, \beta _i, \lambda , i=1\dots ,n$ and obtain the following necessary conditions for optimality:

Stationarity:

$$\begin{aligned}&\nabla _p\frac{\mathbb {P}\left[ {Y=c}\right] }{\mathbb {P}\left[ {Y=c-1}\right] } -\sum _i\alpha _i\nabla _p(p_i^*) + \sum _i\beta _i\nabla _p(p_i^*-1) \\&\qquad + \lambda \nabla _p(\sum _ip_i^*-c +\alpha )=0 \\&\quad \Leftrightarrow \nabla _{p_i}\frac{\mathbb {P}\left[ {Y=c}\right] }{\mathbb {P}\left[ {Y=c-1}\right] } = \alpha _i - \beta _i + \lambda ,~ i=1,\dots , n \end{aligned}$$

The feasibility conditions are the primal constraints, together with the nonnegativity of the multipliers: $\alpha _i,\beta _i \ge 0, i=1,\dots , n$.

Finally, the complementary slackness conditions are:

$$\begin{aligned} -\alpha _ip_i = 0&i=1,\dots , n \\ \beta _i(p_i -1) = 0&i=1,\dots , n \end{aligned}$$

Without loss of generality we consider $p_i>0$ for all *i*. Otherwise, we would just be dealing with a case for smaller *n*. From the complementary slackness conditions we have that $\alpha _i=0$ for all *i*. We consider the following cases.

- $$p_i=1$$.
- $p_i\ne 1$. This implies that $\beta _i=0$. Since $\alpha _i=0$, the stationarity condition states that
  $$\begin{aligned} \nabla _{p_i}\frac{\mathbb {P}\left[ {Y=c}\right] }{\mathbb {P}\left[ {Y=c-1}\right] } = \lambda . \end{aligned}$$
  By lemma 3, we have the following implication.
  $$\begin{aligned} \nabla _{p_i}\frac{\mathbb {P}\left[ {Y=c}\right] }{\mathbb {P}\left[ {Y=c-1}\right] } = \nabla _{p_j}\frac{\mathbb {P}\left[ {Y=c}\right] }{\mathbb {P}\left[ {Y=c-1}\right] }\Rightarrow p_i=p_j. \end{aligned}$$

We thus have that if $\mathbf {p}$ is a minimizer, then for all *i* it is either $p_i=1$ or $p_i=C$ for some constant *C*. The optimal probability vector is therefore of the form

$$\begin{aligned} \mathbf {p} = (\underbrace{1,\dots , 1}_t, \underbrace{\frac{c-t-\alpha }{n-t}, \dots , \frac{c-t-\alpha }{n-t}}_{n-t}) \end{aligned}$$

for some $0\le t < c$. The ratio of probabilities we are trying to bound can be expressed in simple analytical form for vectors of this class:

$$\begin{aligned} \frac{\mathbb {P}\left[ {Y=c}\right] }{\mathbb {P}\left[ {Y=c-1}\right] }&= \frac{{n-t \atopwithdelims ()c-t} \left( \frac{c-t-\alpha }{n-t} \right) ^{c-t}\left( 1-\frac{c-t-\alpha }{n-t} \right) ^{n-c}}{{n-t \atopwithdelims ()c-t-1} \left( \frac{c-t-\alpha }{n-t} \right) ^{c-t-1}\left( 1-\frac{c-t-\alpha }{n-t} \right) ^{n-c +1}} \\&= \frac{(n-c +1) \left( \frac{c-t-\alpha }{n-t} \right) }{(c-t) \left( 1-\frac{c-t-\alpha }{n-t} \right) } \\&= \frac{(n-c +1) \left( c-t-\alpha \right) }{(c-t) \left( n-c +\alpha \right) } \end{aligned}$$

It is easy to see that this decreases in *t* and *n*, and is minimized for $t=c-1, c =1$ at $1-\alpha$. This concludes the proof. $\square$

### Proof of Theorem 3

To prove this theorem we consider two separate cases, which simplifies the analysis. We consider that the events $X_i=1, X_j=1$ happen with probabilities $z_i$ and $z_j$ respectively.

*Case* $$z_i+z_j\ge 1$$:

#### Lemma 4

Let $X=\sum _i x_i$ be a Poisson-Binomial variable with probabilities $\mathbf {z}=(z_1, \dots , z_n)$ and expectation $\mathbb {E}\left[ {X}\right] =k$, for some $k\in {\mathbb {N}}$. Assuming $z_i+z_j\ge 1$ we have

$$\begin{aligned} \frac{{\mathbb {P}\left[ {X=k \bigm | X_i=1, X_j=1}\right] }}{\mathbb {P}\left[ {X=k}\right] } \le \frac{27}{20} \end{aligned}$$

#### Proof

We write $z_i+z_j=1+\alpha$, so that $\mathbb {E}\left[ {Y}\right] = k-1-\alpha$. We can thus apply Theorem 2 to obtain $\frac{\mathbb {P}\left[ {Y=k-1}\right] }{\mathbb {P}\left[ {Y=k-2}\right] } \ge 1-\alpha = 2-z_i-z_j$. We can thus state

$$\begin{aligned} \frac{{\mathbb {P}\left[ {X=k}\right] }}{\mathbb {P}\left[ {X=k \bigm | X_i=1, X_j=1 }\right] } \ge z_iz_j + \frac{(z_i(1-z_j)+(1-z_i)z_j)\mathbb {P}\left[ {Y=k-1}\right] }{\mathbb {P}\left[ {Y=k-2}\right] } \\\ge z_iz_j + (z_i(1-z_j)+(1-z_i)z_j) (2-z_i-z_j), \end{aligned}$$

where the first inequality holds because all terms in the sum are non-negative.

Therefore, in order to prove this result it suffices to solve the following constrained minimization problem.

$$\begin{aligned} \min _{z_i,z_j} \quad&z_iz_j +(z_i(1-z_j) + (1-z_i)z_j)(2-z_i-z_j) \\ \text {subject to } \quad&z_i+z_j \ge 1 \\ \quad&0 \le z_i,z_j \le 1 \end{aligned}$$

By computing KKT points and evaluating the expression at them, we obtain that the minimum value is attained when $z_i=z_j=\frac{2}{3}$, yielding the following bound:

$$\begin{aligned} \frac{\mathbb {P}\left[ {X=k}\right] }{\mathbb {P}\left[ {X=k \bigm | X_i=1, X_j=1}\right] } \ge \frac{20}{27}. \end{aligned}$$

$$\square $$

*Case* $z_i+z_j< 1$: The previous analysis is also helpful in treating this case. Note that the case where we condition on a single variable is the same as in Lemma 4.

#### Lemma 5

Let $X=\sum _i x_i$ be a Poisson-Binomial variable with probabilities $\mathbf {z}=(z_1, \dots , z_n)$ and expectation $\mathbb {E}\left[ {X}\right] =k$, for some $k\in {\mathbb {N}}$. Assuming $z_i+z_j < 1$ we have

$$\begin{aligned} \frac{\mathbb {P}\left[ {X=k \bigm | x_i=1, x_j=1}\right] }{\mathbb {P}\left[ {X=k}\right] } \le \frac{64}{37} \le 1.73. \end{aligned}$$

#### Proof

To prove this bound we use two facts:

- First, the mode of a Poisson-Binomial distribution differs from the mean at most by 1 (Darroch et al. 1964). Since $\mathbb {E}\left[ {Y}\right] > k-1$, we thus have that $\mathbb {P}\left[ {Y=k-1}\right] \ge \mathbb {P}\left[ {Y=k-2}\right]$.
- Second, by Corollary 1 we have $\frac{\mathbb {P}\left[ {Y=k}\right] }{\mathbb {P}\left[ {Y=k-2}\right] } \ge (1-\alpha )^2$.

We thus have a lower bound given by

$$\begin{aligned} \frac{\mathbb {P}\left[ {X=k}\right] }{\mathbb {P}\left[ {X=k \bigm | X_i=1, X_j=1}\right] }&\ge z_iz_j + (z_i(1-z_j)+(1-z_i)z_j) \\+&(1-z_i)(1-z_j)(1-z_i-z_j)^2, \end{aligned}$$

which is minimized at $37/64 \approx 1/1.73$. $\square$

We finally bound the terms involving densities conditioned on a single event.

#### Lemma 6

Let $X=\sum _i x_i$ be a Poisson-Binomial variable with probabilities $\mathbf {z}=(z_1, \dots , z_n)$ and expectation $\mathbb {E}\left[ {X}\right] =k$, for some $k\in {\mathbb {N}}$. Then

$$\begin{aligned} \frac{{\mathbb {P}\left[ {X=k \bigm | X_i=1}\right] }}{\mathbb {P}\left[ {X=k}\right] } \le \frac{4}{3}. \end{aligned}$$

$$\square $$

#### Proof

Now, it is easy to see that

$$\begin{aligned} \frac{\mathbb {P}\left[ {X=k}\right] }{\mathbb {P}\left[ {X=k \bigm | X_i=1}\right] } = z_i+\frac{(1-z_i)\mathbb {P}\left[ {X=k\bigm | X_i=1}\right] }{\mathbb {P}\left[ {X=k-1 \bigm | X_i=1}\right] }. \end{aligned}$$

Applying Theorem 2 again with $Y=(X \bigm | X_i=1)$, we can bound the reciprocal by solving the following constrained minimization problem.

$$\begin{aligned} \min _{z_i} \quad&z_i +(1-z_i)^2 \\ \text {subject to } \quad&0 \le z_i \le 1, \end{aligned}$$

which attains a minimum of $\frac{3}{4}$ at $z_i=\frac{1}{2}$. $\square$

Now, Theorem 3 follows trivially from the combination of lemmas 4, 5 and 6 . $\square$

#### Proof of Theorem 5

Let $X_i=\mathbf {x}_i^T\mathbf {W} \mathbf {x}_i + \mathbf {c}^T\mathbf {x}_i$ be the resulting objective function obtained by the output of the rounding procedure. By Markov’s inequality we know that

$$\begin{aligned} \mathbb {P}\left[ {X_i \ge (1+\epsilon )\mathbb {E}\left[ {X_i}\right] }\right] \le (1+\epsilon )^{-1}. \end{aligned}$$

Thus, if we define the set $S=\{X_1, \dots , X_m\}$, whose elements are the resulting objective function of *m* independent runs of the rounding procedure until a feasible solution is obtained, then with probability at least $1-(1+\epsilon )^{-m}$, there exists at least one *i* such that $X_i \le (1+\epsilon )\mathbb {E}\left[ {X_i}\right] =1.73(1+\epsilon )(\mathbf {z}^T \mathbf {W} \mathbf {z} + \mathbf {c}^T\mathbf {z})$. Thus, if we set $m=\frac{\log (\delta ^{-1})}{\log (1+\epsilon )} = \mathcal {O} \left( \frac{\log (\delta ^{-1})}{\epsilon }\right)$ (for small enough $\epsilon$), and combine this with Lemma 1, we obtain the desired result. $\square$

## Practical implementation

We demonstrate how to solve Problem MinSumSim in practice. Recall that the input matrix $\mathbf {W}'$ is positive semidefinite. Thus, we have

$$\begin{aligned} \mathbf {x}^T \mathbf {W}' \mathbf {x} = \Vert \mathbf {Q}\varvec{\Sigma }^{1/2} \mathbf {x} \Vert ^2, \end{aligned}$$

where $\mathbf {W}'=\mathbf {Q}\varvec{\Sigma }\mathbf {Q}^T$ is the eigendecomposition of $\mathbf {W}'$. This is useful because convex programming solvers can often handle input in the form $\mathbf {Q}\varvec{\Sigma }^{1/2}$. If the numerical rank of $\mathbf {W}'$ is small, this representation will be much more memory-efficient than $\mathbf {W}'$. Furthermore, we can approximate $\mathbf {W}'$ using a low-rank approximation. Aside from memory efficiency, this avoids convexity tests implemented by some solvers, which suffer from numerical instability.

Perhaps more importantly, in practical settings we are usually given a *data matrix* $\mathbf {D} \in {\mathbb {R}}_+^{n\times m}$, consisting of document vectors instead of a similarity matrix, with $n\gg m$ (if $n\approx m$, it is usually possible to reduce the dimensionality with little loss in accuracy). If using, say, cosine similarities, we do not need to build the matrix $\mathbf {W}'$ explicitly, as providing $\mathbf {D}$ to a solver will suffice.

## Randomized rounding algorithms

*Dependent rounding* We implement the depedendent randomized rounding algorithm described by Srinivasan (Srinivasan 2001). The algorithm works by picking a pair of variables, rounding at least one of them, and deferring the remaining one (if any) to the next step (see the simplify procedure in the cited work (Srinivasan 2001)). Once all variables have been treated, the procedure is repeated on the ones not yet rounded. This goes on until an integral solution is obtained. This results in a binary tree, each of whose levels must be processed sequentially. As described by Doerr and Wahlström (Doerr and Wahlström 2015) we randomize the order of the variables in *z* to ensure the quality of the rounded solutions. We emphasize that before we implemented this step, this algorithm produced unsatisfactory results in some data sets. We then collect $\log (\delta ^{-1})/{\epsilon }$ feasible solutions, which is roughly the number required by our independent method (see Theorem 5).

*Independent rounding* For the independent randomized algorithm, we generate a random matrix of $\sqrt{k}\log ^2(\delta ^{-1})/{\epsilon }$ rows and *n* columns. We obtain integral solutions by rounding each row of random variables against *z*. These rounded solutions need not be feasible wrt the cardinality constraint of *k*. Therefore, we compute the row sums and return the first $\log (\delta ^{-1})/{\epsilon }$ feasible solutions.

To optimize the space requirements, we do not instantiate the random matrix. Instead we generate individual random numbers in parallel and proceed to immediately round it. This reduces the space requirement from 64 bits for a random number to just 8 bits to store a rounded variable. Further space optimizations are possible, as each rounded variable requires only 1 bit. We experimentally observed that our implementation produces, on average, twice as many feasible solutions as expected. This suggests that our bounds are slightly pessimistic (see Theorem 5), which stems in part from the use of the Binomial density as a lower bound in Lemma 1.

To make sure that the observed running times are not highly dependent on the chosen programming language, we implemented both randomized rounding algorithms in C**++** utilizing Cython (Behnel et al. 2011). While this produced a ten fold improvement in running time for both algorithms, the overall trends remain similar, as seen in Fig. 8 (bottom). The objective ratios (top) are similar, and the differences are likely due to the random elements inherent to the experimental setup.

## Additional information on the datasets

We select publicly available collections of documents resulting from queries. Since each document is query-dependent, and the same document could be returned by multiple queries (with some varying query-dependent features), we consider query-document pair to be unique. We consider the following datasets:

Letor (Learning to rank) is a package of benchmark information-retrieval datasets. LETOR4.0 (Qin and Liu 2013) contains two query sets from Million Query track of TREC 2007 and TREC 2008, which we call MQ2007 and MQ2008. Each document comprises 46 features such as TF-IDF, BM25 and LMIR and the relevance scores are 0, 1 and 2. We also consider one dataset (Gov2003hp) from the Web Track of TREC 2003 and TREC 2004 (LETOR3.0 (Qin et al. 2010)), an information-retrieval task on a crawl of a .gov domain, with three tasks: homepage finding (hp), name page finding (np), and topic distillation (td). Similarly to LETOR 4.0, each document comprises 64 features such as TF-IDF, BM25 and LMIR.

Census (Dua and Graff 2017) is a dataset extracted from the 1990 US census. It contains $2\,458\,285$ documents, each with 68 demographic features.

Istella (Istella extended learning to rank) (Lucchese et al. 2018) is composed of 10 000 queries and 220 features representing each query-document pair. Istella-X LETOR consists of 26 791 447 pairs produced by retrieving up to 5 000 documents per query according to the BM25F ranking score. It has been split to train, validation and test sets according to a 60–20–20% scheme. We use the validation set.

## Footnotes

1 https://version.helsinki.fi/mahadeva/diversified-item-selection-experiments.
